# A Comparative Study of the Self-Cleaning and Filtration Performance of Suspension Plasma-Sprayed TiO_2_ Ultrafiltration and Microfiltration Membranes

**DOI:** 10.3390/membranes13090750

**Published:** 2023-08-23

**Authors:** Elnaz Alebrahim, Christian Moreau

**Affiliations:** Department of Mechanical, Industrial, and Aerospace Engineering, Concordia University, Montreal, QC H3G 1M8, Canada; elnazsadat.aleebrahim@mail.concordia.ca

**Keywords:** suspension plasma spray, ceramic membrane, ultrafiltration, microfiltration, fouling, self cleaning

## Abstract

This study investigated the performance of photocatalytic titanium dioxide microfiltration membranes with an average pore size of approximately 180 nm and ultrafiltration membranes with an average pore size of around 40 nm fabricated with the suspension plasma spray process. The membranes were evaluated for their filtration performance using SiO_2_ particles of different sizes and polyethylene oxide with molecular weights of 20 kDa to 1000 kDa, and the fouling parameters were characterized. The rejection rate was enhanced by increasing the thickness of the membranes. This effect was more pronounced with the ultrafiltration membranes. The rejection rate of the ultrafiltration membrane was improved significantly after filling the larger pores on the surface with agglomerates of titanium dioxide nanoparticles. The self-cleaning performance of the membranes was assessed under visible light. Both ultrafiltration and microfiltration membranes showed a flux recovery under visible light illumination due to the photocatalytic activity of titanium dioxide. The membranes also show a flux recovery of more than 90%.

## 1. Introduction

Over the past few decades, the significant expansion of industrialization, coupled with climate change, population growth, and water contamination has emerged as a critical concern for sustainable development due to the increasingly severe scarcity of water resources [1,2]. Therefore, affordable and highly efficient membrane-based technologies with low energy consumption, a modular nature, and a small footprint have become increasingly popular for water and wastewater treatment [3,4]. Among these technologies, microfiltration (MF) membranes with a pore size ranging from 100 nm to 10 µm and ultrafiltration (UF) membranes with a pore size of 2–100 nm are widely utilized due to their ability to remove contaminants with large molecules at low pressure [5].

Due to their superior mechanical strength, resistance to fouling, and high chemical and thermal resistance, ceramic membranes are regarded as favorable candidates for water treatment [6]. However, when it comes to the extensive utilization of ceramic membranes, capital cost and efficiency are critical considerations and present notable challenges. To overcome these challenges, new materials, production processes, and performance-improving techniques have been explored [7]. Despite the numerous advantages offered by the MF and UF processes in membrane technology, the challenge of membrane fouling persists and influences the development of membrane applications [8]. The performance of the membranes can be easily affected by fouling, which may occur from organic and inorganic substances and bio-contaminants [9,10]. Subsequently, fouling causes a reduction in membrane flux, separation efficiency, and lifespan while also increasing cost and energy consumption [10,11]. Various approaches have been employed to mitigate membrane fouling, including physical and chemical cleaning, pretreatment of the feed, combining membrane technologies and processes, and the development of anti-fouling and self-cleaning membranes [8,10]. Typically, the fouling caused by organic colloids on membranes is addressed by pretreating the feed and optimizing process parameters. Therefore, the development of self-cleaning and anti-fouling membranes is focused on reducing the fouling effect of organic contaminants [10]. Integrating photocatalysis properties into the filtration process is an ever-growing approach that could enhance the purification efficiency of the membrane by providing a self-cleaning potential. Titanium dioxide (TiO_2_) is a semiconductor that can degrade organic contaminants due to photocatalytic reactivity. When exposed to UV light, the photogenerated electron–hole pair may react with water and oxygen to produce highly oxidizing reactive oxygen species (ROSs) such as HOO˙, O_2_˙^−^, and ˙OH radicals. Further, ROSs contribute to the decomposition of organic contaminants [4,12]. Humic acid (HA) is a model organic contaminant widely used to characterize membrane processes [10]. Humic substances, including humic acids, are formed through the natural processes of the microbiological or chemical decomposition of organic matter and are found in soil and surface water [13,14]. HA is substantially used in the fertilizer production industry. The presence of a high dosage of HA in water could result in the generation of toxic material during the chlorination process [15]. Thus, removing HA is essential to the water treatment process before disinfection.

The rejection properties and anti-fouling/self-cleaning of the TiO_2_ incorporated membranes have been discussed in many studies. Teow et al. [16] investigated the influence of surface morphology on the anti-fouling property of PVDF/TiO_2_ membranes against HA under UVA illumination, with an enhanced flux recovery for the membranes with lower surface roughness. Zhang et al. [17] investigated the influence of hydrogenation on the HA removal efficiency of TiO_2_ membranes prepared via spinning and partial sintering processes under UVA light. Li et al. [18] studied the influence of surface modification of ceramic membranes coated with a TiO_2_-GO composite on photocatalytic reactivity to remove HA, where pore blocking was the primary fouling mechanism. Lin et al. [19] characterized the fouling mechanisms in TiO_2_ MF membranes produced via the atmospheric plasma spray (APS) process using silicon dioxide (SiO_2_), HA, and dextran. In their study, pore blocking and cake formation resistance were the dominant mechanisms influencing the photolysis filtration of HA and dextran solutions.

Recently, suspension plasma spray (SPS) technology was introduced by the authors as a novel method to fabricate TiO_2_ membranes with average pore sizes in the range of MF and UF, with a rather high pure water permeability and photocatalytic activity under UV and visible light [20,21]. SPS is a developing thermal spray coating deposition process that can produce thin nanostructured coatings due to using a feedstock of submicron- to nanometer-sized particles suspended in a solvent. In SPS, the suspension of very fine particles is injected in a high-temperature and high-velocity plasma jet, where the temperature in the core of the plasma jet exceeds 8000 K [22]. Due to the aerodynamic drag forces in the plasma jet, the suspension droplets break into smaller fragments and are accelerated toward the surface of a substrate [23]. In an ideal process, when all the fragmentation occurs close to the plasma core, the solvent that carries the inflight particles may evaporate completely, and the particles melt before impacting the substrate surface and are deposited in the form of splats [24]. However, the inflight particles that travel in the fringes of the plasma, gaining lower temperature and momentum, may be partially melted or remain unmelted [22]. Subsequently, the coating is formed layer by layer through the repeated impact of the melted, partially melted, and unmelted particles onto the substrate. In addition, the thickness of the SPS coating can be adjusted by changing the number of times the plasma torch scans the surface of the substrate. Furthermore, coatings with various microstructures can be produced by adjusting the feedstock properties and the spray parameters. These microstructures can vary from fully dense coatings [24] to porous coatings with vertical cracks [25], porous coatings with various forms of columnar features that appear in the form of bumps on the surface [25,26,27,28], and uniformly porous coatings [26]. This versatility allows the production of coatings suitable for a wide range of applications.

SPS membranes own a unique porous microstructure, where the porosity depends on the presence of the retained unmelted feedstock particles within the structure, and the average pore size relates to the particle size of the pristine feedstock powder. Additionally, SPS TiO_2_ membranes exhibit photocatalytic properties under UV and visible light [20,21]. The photocatalytic activity of TiO_2_ membranes under visible light is due to the generation of substoichiometric TiO_2−x_ under SPS conditions [27]. The presence of oxygen vacancies in the lattice of TiO_2−x_ in the membrane, known as self-doping, leads to lower bandgap energy [21]. Consequently, SPS TiO_2_ membranes can absorb lower-energy photons in the range of visible illumination. Visible-light-driven photolysis could introduce a promising alternative to decrease the energy requirement for UV utilization.

Other advantages of the SPS method over conventional ceramic membrane fabrication techniques and other thermal spray processes could be outlined as flexibility and efficiency, which align well with industrial requirements [28]. The primary emphasis of our earlier works was to investigate the feasibility of creating porous structures with pore sizes in the range of UF and MF membranes suitable to use as filtration membranes. [20,21]. In this study, we aimed to build upon those previous efforts and delve deeper into the performance aspects of the SPS membranes. To better understand the benefits and limitations of SPS ceramic membranes, this work investigates the filtration and self-cleaning characteristics of the SPS MF and UF membranes produced in our previous works. The inorganic contaminants (SiO_2_ particles) were used to characterize the rejection efficiency of the SPS membranes. In addition, organic pollutants (humic acid (HA) and methylene blue (MB)) were used to study the self-cleaning performance and recyclability of the membranes. Furthermore, the fouling mechanisms of the model contaminants were characterized.

## 2. Materials and Methodology

### 2.1. Membrane Preparation

The suspension plasma spray (SPS) process was used to produce the TiO_2_ membranes. The membranes were sprayed on porous stainless-steel discs (diameter: 38 mm, thickness: 1.6 mm). The thickness of the membranes was controlled by changing the number of times the plasma torch passed over the surface of the stainless-steel substrates (spray passes).

The membranes were identified based on the number of spray passes, according to Table 1. Membranes UF-2×4P and MF-2×12P were built by stacking two UF-4P and two MF-12P, respectively. The stacked configuration was achieved by placing one membrane over another and securing them together using silicon glue.

The details of the fabrication process for the SPS microfiltration (MF) membranes [20] and ultrafiltration (UF) membranes [21] and comprehensive discussions regarding the microstructures and some particular properties of the UF and MF membranes have been published in our previous works. For a clearer understanding and to contextualize the findings in this current manuscript, here, a concise overview of the UF and MF membrane characteristics based on our previous research is presented.

The UF membranes were produced using a nanosized TiO_2_ powder. The average TiO_2_ particle size was 27 ± 10 nm. However, the TiO_2_ nanosized particles formed natural agglomerates with d_50_ = 3.1 µm in the feedstock suspension used in the SPS process. The presence of these agglomerates proved to be essential to produce nanosized pores within the structure [21]. On the other hand, the MF membrane was produced using a submicron-sized TiO_2_ powder, where the average TiO_2_ particle size was 137 ± 40 nm, and the SPS feedstock suspension showed d_50_ = 280 nm [20]. The average pore size and the pore size distribution of the UF and MF membranes were obtained using the mercury intrusion porosimetry method. The average pore size of the UF membranes was around 36 nm, which was in the range of ultrafiltration. However, the pore size exhibited a multi-modal distribution due to the presence of some large pores in the range of a few micrometers [21]. Alternatively, the average pore size of the MF membranes was around 180 nm, with a relatively narrow pore size distribution [20]. A brief description of some of the features of the SPS membranes can be found in the Supplementary Materials section.

### 2.2. Membrane Characterization

The surface roughness of the membranes was measured with a confocal laser microscope (LEXT OLS4000 Olympus, Toronto, ON, Canada). Three-dimensional surface images were obtained by stitching 25 single images of the surface in three spots. The arithmetic average surface roughness (R_a_) and the mean height difference between the highest peak and five lowest valleys (R_z_) [29] have been reported. In addition, the morphology of polished cross-sections of the membranes was observed with an ultra-high-resolution scanning electron microscope (SEM) (Hitachi Regulus 8230, Mississauga, ON, Canada). 

The water contact angles on the surface of the membranes were obtained using a contact angle measuring system (VCA, AST products Inc., Billerica, MA, USA). 

### 2.3. Membrane Performance

The separation efficiency of the membranes was determined using 1 wt.% aqueous colloidal suspensions of SiO_2_ (PiKem Co., Tamworth, UK) with average particle diameters of 200 and 400 nm. The particle size distribution of the two SiO_2_ powders was measured using a Zetasizer Nano ZS system (Malvern Panalytical, Malvern, UK). It was noted that the SiO_2_ powder with an average particle size of 200 nm displayed a broader particle size distribution (Supplementary Materials, Figure S1).

The filtration was carried out in a dead-end vacuum filtration device. A mixing device was added to the filtration system to keep the feed agitated during the process. The transmembrane pressure for all the performance measurement tests was around 0.3 bar. The concentration of the SiO_2_ particles in the filtrate and feed was measured using a turbidity meter (Oakton T-100, Cole-Palmer, Quebec, QC, Canada). It has been reported that turbidity is directly related to the total suspended solid concentration of slurries [30,31]. Calibration curves were produced to obtain the concentrations of suspended SiO_2_ in the filtrate. In both 200 nm and 400 nm SiO_2_ suspensions, the concentration showed linearity with the turbidity. The concentration of the SiO_2_ particles in the filtrate was measured by collecting about 10 mL of filtrate after around 30 min of filtration.

The separation efficiency was calculated using Equation (1) [32]:

(1) $$R\;\left(\%\right)=\left(1-\frac{C_{f}}{C_{0}}\right)\times 100$$

where *C_f_* and *C*_0_ are the concentration of the contaminant in the filtrate and feed, respectively.

The permeation flux was calculated using Equation (2) [16]:

(2) $$J=\frac{V}{A\cdot t}$$

where *J* is the permeation flux (L·m^−2^·h^−1^), *V* is the permeation volume (L), *t* is the permeation time (h), and *A* is the surface area of the membrane (m^2^). 

The flux recovery ratio (FRR) was calculated using Equation (3) [32,33]:

(3) $$\mathrm{FRR}\;\left(\%\right)=\frac{J_{c}}{J_{0}}\times 100$$

where *J_c_* is the permeation flux of DI water after backwashing or self-cleaning (L·m^−2^·h^−1^), and *J*_0_ is the initial permeation flux of DI water (L·m^−2^·h^−1^). The membranes were backwashed with 1000 mL of DI water at the transmembrane pressure of 0.3 bar. 

The analysis of the fouling characteristics of the membranes during the SiO_2_ particle separation was conducted by evaluating the ratio of fouling resistance (RFR) using Equations (4)–(6) [16,33,34]:

(4) $$RFR_{t}\left(\%\right)=\left(1-\frac{J_{t}}{J_{0}}\right)\times 100$$

(5) $$RFR_{P}\left(\%\right)=\left(1-\frac{J_{p}}{J_{0}}\right)\times 100$$

(6) $$RFR_{C}\left(\%\right)=RFR_{t}\left(\%\right)-RFR_{p}\left(\%\right)$$

where *RFR_t_* is the total ratio of fouling resistance due to pore blocking and cake formation. *J_t_* is the flux of the SiO_2_ suspension (L·m^−2^·h^−1^), *J*_0_ is the initial permeation flux of DI water (L·m^−2^·h^−1^), *RFR_p_* is the ratio of fouling resistance due to pore blocking, *J_P_* is the DI water flux measured after removing the SiO_2_ cake layer (L·m^−2^·h^−1^), and *RFR_c_* is the ratio of fouling resistance due to cake formation.

The SPS TiO_2_ membranes exhibited absorbance and photocatalytic activity under visible light [21]. Therefore, the self-cleaning performance and recyclability of the membranes were investigated in three cycles using a dead-end vacuum filtration device under visible-light conditions [32]. The surface of the membrane was illuminated with two xenon arc lamps with a power of 35 W, each with an irradiance of 2.16 mWcm^−2^. No optical filter was used. The distance between the surface of the membrane and the light source was set at around 5 cm, and an electric fan was used to cool the setup. A 2 ppm solution of humic acid (68131-04-4, Sigma-Aldrich, St. Louis, MO, USA) with the pH adjusted to around seven was used to characterize the membranes’ self-cleaning performance [16,19]. Each cycle consisted of three steps: (i) filtering DI water for 15 min in the dark, (ii) filtering HA solution for 15 min in the dark, and (iii) filling with DI water and exposing the surface of the membranes to visible light for 150 min with no pressure. The evolution of the normalized flux was monitored as an indicator of the self-cleaning property of the membranes. The volume of the HA solution was maintained at around 30 mL throughout the duration of the experiment. In order to evaluate the extent of flux recovery after the self-cleaning process, following the 3rd cycle, around 30 mL of DI water was added to the system, and the membranes were exposed to visible light for 4 h and then were backwashed with 1000 mL of DI water and rinsed thoroughly with DI water. The membrane DI water flux was measured after the 4 h exposure to visible light and after the hydraulic cleaning. Additionally, the recyclability of the UF-2P and UF-12P membranes in terms of the photodegradation of the dye solution was investigated in three static cycles of 120 min using a 6 ppm methylene blue (MB) solution. After each cycle, the membrane was cleaned by immersing it in ethanol for around 12 h on the magnetic stirrer, flushing and rinsing thoroughly with DI water, and exposing it to UVC light for 12 h. The details of the test and the experimental setup for the MB degradation experiment have been described elsewhere [21]. 

The molecular weight cutoff (MWCO) was determined using 1 wt.% polyethylene oxide (PEO) with molecular weights of 20, 100, 300, and 1000 kDa obtained from Sigma-Aldrich. The concentration of PEO was measured with a UV–vis spectrophotometer (Cary 8454, Agilent, Mississauga, ON, Canada) at the fixed wavelength of 535 nm [35,36]. For PEO, the molecular weight (MW) was converted into the pore diameter (nm) using Equation (7) [37]:

(7) $$d_{50}=0.11\;MW_{50}{}^{0.46}$$

where *MW*_50_ indicates the molecular weight of the organic molecule that can be rejected by 50%.

The Stokes radius of PEO was obtained using Equation (8) [38]:

(8) $$a=10.44\times 10^{-3\;}{\left(MW\right)}^{0.587}$$

where *a* is the Stokes radius (nm), and *MW* is the molecular weight of the PEO.

This study also utilized an infiltration process of nanoparticles into the structure of UF membranes to explore potential enhancements in the performance of UF membranes. Therefore, the manuscript presents two sets of results: first, the results for the untreated membranes, referred to as “as-sprayed” membranes, and following that, from Section 3.3, the results obtained for the membranes subjected to infiltration, referred to as “filled membranes.” This paper discusses the outcomes obtained from both sets of membranes in detail.

## 3. Results and Discussion

### 3.1. Membrane Microstructure and Roughness (As-Sprayed Membranes)

**Figure 1:**
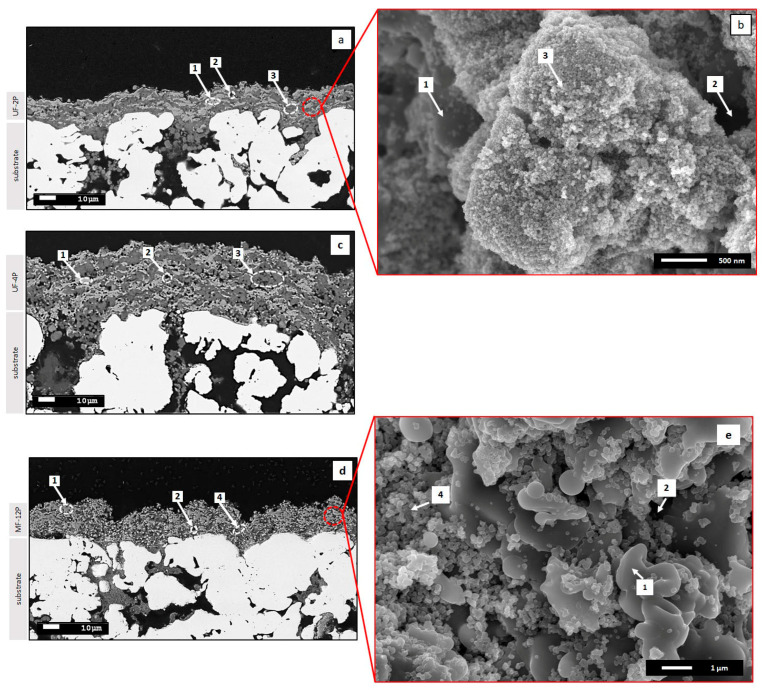
(**a**) SEM micrograph of the cross-section of the U-2P membrane, (**b**) high-magnification SEM micrograph of the red dashed zone in UF-2P membrane, (**c**) SEM micrograph of the cross-section of the U-4P membrane, (**d**) SEM micrograph of the cross-section of the MF-12P membrane, and (**e**) high-magnification SEM micrograph of the red dashed zone in MF-12P membrane. In both UF and MF membranes, no. 1 (light grey areas) shows the melted splats, no. 2 (black areas) shows the large pores, no. 3 (dark grey areas) shows the agglomerates of unmelted nanosized TiO_2_ particles in the UF membrane, and no. 4 (dark grey areas) shows the unmelted submicron-sized TiO_2_ particles in the MF membrane.

Figure 1 illustrates the SEM micrographs of the UF-2P, UF-4P, and MF-12P membranes. An overview of the cross-sections of UF-2P, UF-4P, and MF-12P membranes are shown if Figure 1a, Figure 1c and Figure 1d, respectively. The main features of the SPS membranes include (1) light grey areas made of the melted splats, (2) black areas, which are the large pores in between the randomly stacked melted splats and/or agglomerates of nanosized TiO_2_ particles, and dark grey areas, which in UF membranes are made of (3) agglomerates of unmelted nanosized TiO_2_ particles, and in MF membranes are made of (4) unmelted submicron-sized TiO_2_ particles. The dark grey zones in the UF and MF membranes are finely porous regions. In the UF membranes, these pores are of nanoscale size and result from unmelted nanosized TiO_2_ particles deposited as micron-sized agglomerates. In contrast, the dark grey regions in the MF filtration membranes exhibit submicron-sized porosity, which arises from the gaps between unmelted submicron-sized TiO_2_ particles. Additionally, the few micron-sized pores in the UF membranes, which closely aligned with the average size of the agglomerates of nanosized particles in the feedstock suspension, were linked to the large inter-agglomerate and inter-splat gaps, indicated by no. 2, observed in Figure 1a and Figure 1c [21]. Figure 1b,e provide a detailed representation of the regions described earlier, namely regions 1 to 4. The microstructures of the UF-2P and UF-4P membranes shared similar detailed features. Thus, it is expected that the detailed features presented in Figure 1b for UF-2P are consistent in UF-4P. A comprehensive discussion regarding the microstructures of the UF and MF membranes produced with the SPS process can be found elsewhere [20,21].

Table 2 displays the thickness of the UF and MF membranes. The thicknesses of UF-2×4P and MF-2×12P were estimated to be twice those of UF-4P and MF-12, respectively.

**Figure 2:**
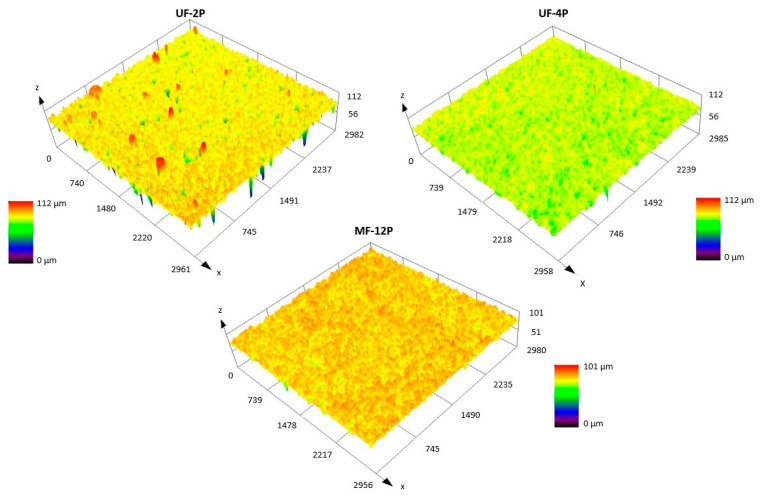
Confocal microscope images of UF-2P, UF-4P, and MF-12P membranes.

The confocal microscope images of the UF-2P, UF-4P, and MF-12P membranes in Figure 2 reveal the surface roughness of these membranes, while Table 3 provides the corresponding R_a_ and R_z_ roughness values. It is evident that the UF membranes have a lower roughness compared to the MF membranes. Furthermore, within the UF membranes, increasing thickness leads to decreased surface roughness. As mentioned earlier, higher surface roughness could make the membrane more susceptible to fouling. On the other hand, an increased roughness could probably enhance the photocatalytic performance by providing a larger reactive surface area. The higher surface roughness observed in the MF-12P membrane is attributed to the presence of columnar features or bumps on its surface (a brief description can be found in the Supplementary Materials section). Additionally, the larger pores on the surface of the stainless-steel substrate and the presence of smaller inflight species in the MF membrane feedstock, compared to the larger agglomerates in the UF membrane feedstock, could contribute to the higher roughness of the MF membrane.

### 3.2. Membrane Performance (As-Sprayed Membranes)

#### 3.2.1. Separation Performance

**Figure 3:**
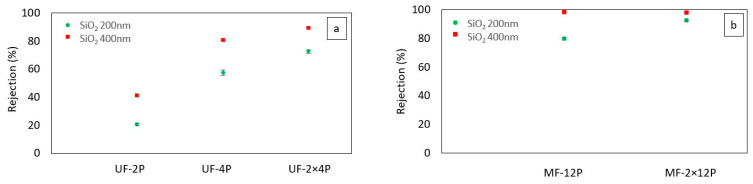
The SiO_2_ particles rejection % of (**a**) UF membranes and (**b**) MF membranes. The separation efficiency was increased as a function of the thickness of the membranes.

Figure 3 shows the particle rejection efficiency of the UF and MF membranes. Both the UF and MF membranes exhibit an enhancement in particle rejection as the membrane thickness increases. Similar findings regarding the impact of membrane thickness on rejection have been reported by Ramakrishnan et al. for thermally sprayed MF membranes [39] and by Ding et al. for TiO_2_ UF membranes fabricated using a wet chemical method [40]. Wang et al. [41] also reported an increase in the rejection efficiency of two-dimensional graphene carbon nitride nanosheet membranes at higher thicknesses. Figure 3 also shows that overall, the MF membranes demonstrate superior particle rejection compared to UF membranes, and specifically, the UF-2×4P membrane exhibits particle rejection rates comparable to the MF-12P membrane. The higher rejection of the MF membrane can be explained by its narrow pore size distribution with an average pore size of around 180 nm [20]. On the other hand, although the average pore size of the UF membrane was about 40 nm, the pore size distribution was multimodal, with some larger pores in the range of a few micrometers corresponding to the inter-agglomerates and inter-splat pores [21]. The presence of large pores can be the reason for the lower particle rejection in the case of UF membranes [42]. Additionally, it is worth noting that the broader range of particle sizes observed in the feed made with SiO_2_ with an average particle size of 200 nm might have played a role in enhancing the separation efficiency of the membranes when that particular feed was used. The improved separation efficiency for feeds containing mixed-molecular-weight solutes could be attributed to pore blocking, where larger particles that can still penetrate the pores may reduce the pore size. Reducing the pore size may result in a higher separation for the smaller particles in the feed [43].

During the filtration process, both UF and MF membranes underwent a substantial flux reduction (Supplementary Materials, Figure S2). Flux decline during filtration occurs due to the fouling of the membranes through pore fouling and cake formation [16,19,32]. Also, employing a dead-end filtration system could have played a role in reducing the flux. By continuing the filtration in the dead-end mode, the thickness of the porous cake layer on the surface increases, leading to a decrease in the filtration rate. The formation of the cake layer may also improve the filtration efficiency. However, after a certain point, the excessive cake thickness interrupts the filtration process and the membranes need to be cleaned [44]. Among the five membranes evaluated, it seems that MF-2×12P demonstrated the most favorable performance, exhibiting the lowest drop in flux and the highest particle rejection rates.

**Figure 4:**
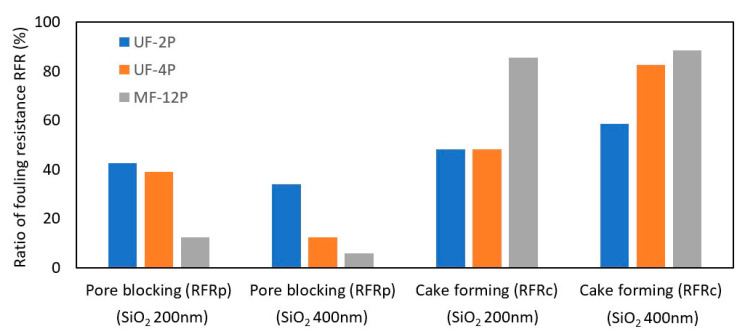
Fouling behavior of UF-2P, UF-4P, and MF-12P due to the pore blocking (obtained from RFRp) and cake formation (obtained from RFRc) phenomena. A lower RFR value indicates a higher fouling resistance.

During the SiO_2_ separation processes, fouling occurs due to two main mechanisms: pore blocking and cake formation. In this work, the ratio of fouling resistance (RFR) was used to characterize the fouling behavior of the membranes. Figure 4 illustrates the fouling behavior of the UF-2P, UF-4P, and MF-12P membranes. In Figure 4, the ratio of pore blocking is represented by the RFR_p_, and the ratio of cake formation is defined by RFR_c_. The RFR was not calculated for the UF-2×4P and MF-2×12P membranes since the cake layer could not be removed effectively from the surface of the stacked samples.

A lower RFR generally means higher fouling resistance [16]. The primary fouling mechanism observed in the MF-12P membrane for both SiO_2_ particle sizes is the formation of a cake layer. Moreover, the MF-12P membrane demonstrates a significantly higher resistance to pore blocking than UF membranes. Furthermore, the findings suggest that increasing the thickness of UF membranes, especially at larger foulant sizes, reduces their tendency for pore blocking fouling. The influence of pore blocking and cake formation seems comparable for the UF membranes at smaller foulant sizes. 

The multimodal nature of the pore size distribution and the presence of some large pores in the UF membranes has probably made them more susceptible to fouling due to pore blocking. While the larger particles are rejected through the sieving mechanism, smaller particles could infiltrate the pores and decrease the pore size through deposition on the pore walls and narrowing the pores [9,45]. Therefore, the UF membranes are more prone to pore blocking fouling than the MF membranes, which have a narrow pore size distribution, and the pore size is close to or smaller than the SiO_2_ particle size. It also could be the potential reason for increased resistance to pore blocking fouling with increasing the thickness of UF membranes. In UF-4P, the narrowing of the pore close to the surface of the thicker UF membrane could probably prevent further fouling by creating a narrower pore size distribution. The fouling mechanism may convert to cake formation following the generation of smaller pores on the surface. The pore blocking fouling is more pronounced in the case of smaller SiO_2_ particle sizes, where a more significant difference exists between the foulant particle size and membrane pore size. Additionally, the flux reduction in the UF membranes could be attributed to their increased tendency for pore blocking, corresponding to their relatively wide pore size distribution. In the SPS UF membranes, the presence of some larger pores contributes to a high initial flux. Consequently, the foulants may penetrate more easily into the pores and cause a more severe flux decline due to the pore blocking phenomenon [46]. These observations may suggest that the average pore size does not solely determine the SiO_2_ particle rejection efficiency of the SPS membrane, and the presence of a narrow pore size distribution also appears to play a significant role in influencing the separation performance [47].

Table 4 summarizes the flux recovery ratio (FRR) of the UF and the MF membranes. The UF-2P, UF-4P, and MF-12P membranes showed an FRR over around 95%. The UF-2×4P and MF-2×12P membranes demonstrated a lower FRR than the other membranes. The UF-2×4P and MF-2×12P membranes were backflushed while preserving their stacked arrangement without any separation. Therefore, the lower FRR of UF-2×4P and MF-2×12P could correspond to less-efficient cleaning through the backwashing process due to the attachment of the membranes. 

#### 3.2.2. Self-Cleaning and Recyclability

**Figure 5:**
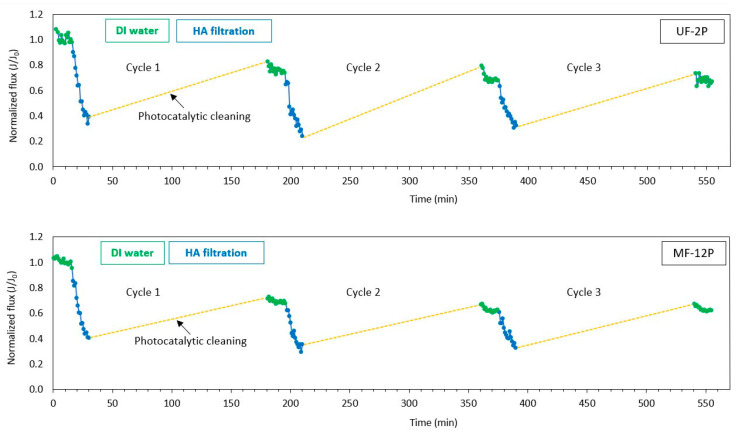
Self-cleaning performance of UF-2P and MF-12P membranes repeated in three cycles showing partial flux recovery of the membranes due to the photocatalytic cleaning of the membranes.

Figure 5 illustrates the self-cleaning performance of the UF-2P and MF-12P membranes. The selection of UF-2P was based on its similarity in terms of thickness and surface roughness to that of MF-12P, allowing a more direct comparison between the two membranes. The self-cleaning trend observed in both the UF-2P and MF-12P membranes was relatively similar during the three cycles of the experiment. Initially, during the filtration stage involving HA, a sharp decline in flux is observed, attributed to membrane fouling. Fouling could occur due to a combined effect of an accumulation of the foulants on the surface of the membranes, forming a cake layer and pore blockage throughout the membrane thickness [19]. 

The self-cleaning property of a membrane is a parameter that can influence the lifespan of the membrane significantly. In this work, a series of dark and photocatalysis experiments were used to study the self-cleaning characteristics of the UF-2P and MF-12P membranes in terms of HA removal. 

After the photocatalytic cleaning in the first cycle, the membranes partially recovered their initial flux, showing the removal of the foulants from the surface. However, the DI water flux does not fully return to its initial value before fouling occurs. This could be attributed to the pore blocking phenomenon due to the wide range of molecular weights in the HA solution [16,19]. Nystrom et al. [48] reported a more significant HA fouling in membranes with larger pores due to pore blocking. Another possibility could be that the foulants were not completely decomposed during the photocatalytic cleaning process. It has been reported that the photocatalytic degradation of HA in the presence of TiO_2_ could initially be hindered due to the photodepolymerization of large aromatic structures in HA. Subsequently, the photodegradation of HA follows pseudo-first-order kinetics, resulting in the mineralization of HA into by-products [49,50]. Additionally, the depolymerization process lowers the molecular weight of the HA. A reduction in the molecule size could result in a decrease in the membrane flux due to narrowing the pore size [45]. On the other hand, smaller molecules can also pass more easily through the pores and potentially contribute to flux recovery as well. Therefore, the observed partial increase in the flux could have probably occurred even without a complete degradation of the foulant. A similar trend was observed during the second and third cycles of the experiments, with a slight decrease in the DI water flux toward the end of the experiment. The flux recovery ratios during the three cycles of experiments were 75%, 68%, and 67% for the UF membrane and 69%, 61%, and 62% for the MF membrane, indicating the recyclability of the de-fouling process. It is also worth noting that the kinetics of the photodegradation of HA by TiO_2_ can be influenced by various factors related to the properties of the HA substance itself. These factors include the origin of the HA, the method of extraction, its interactions with the environment, and age [51]. Furthermore, other parameters, such as the morphological characteristics of TiO_2_ [12] and the chemistry of water [52], and the experimental parameters, such as the wavelength and the intensity of the light, also influence the photocatalytic degradation process.

The photocatalytic activity of the SPS TiO_2_ membranes under visible light is due to the generation of oxygen vacancies in the lattice of TiO_2_, known as self-doping. As a result, the band gap energy of the SPS TiO_2_ membrane is reduced, allowing the photogeneration of electron–hole pairs under visible light [21,27].

The photodegradation of HA occurs due to the breakup of large aromatic species followed by mineralization [49,50]. Corin et al. determined the formation of carboxylic acids with lower molecular weight compared to that of HA through the direct photolysis of HA [53]. It has also been reported that in the presence of TiO_2_ as the photocatalyst, the photodepolymerization of the adsorbed HA molecules could occur through successive oxidation of the carboxylate or phenolate surface groups [49]. During the photocatalytic reaction, the photogenerated electron–hole pairs in TiO_2_ form powerful oxidizing species through interaction with water and oxygen. Holes may react with H_2_O and O_2_ to produce H^+^ and ˙OH (hydroxyl radicals). Meanwhile, electrons react with O_2_ to generate O_2_˙^−^, which can further yield ˙OH radicals. Furthermore, hydrolyses of water molecules on the surface of the TiO_2_ photocatalyst lead to the generation of free radicals and ˙OH groups [12]. The highly reactive hydroxyl radicals break down molecules of HA through chemical reactions involving hydroxyl addition or hydrogen extraction and eventually decompose HA or HA intermediate molecules [16].

**Figure 6:**
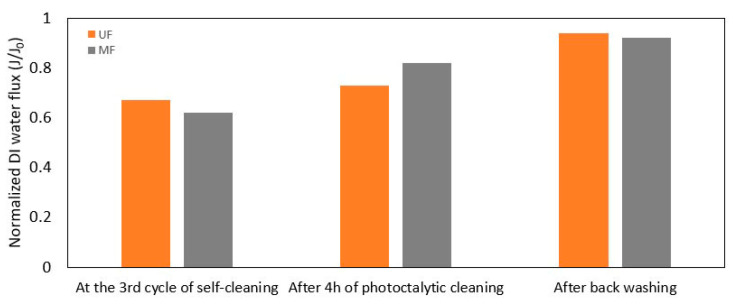
Evolution of the flux recovery of UF-2P and MF-12P membranes after four hours of photocatalytic cleaning and backwashing following the 3rd cycle of the self-cleaning experiment.

After completing the third cycle, the flux recovery of UF-2P and MF-12P membranes was measured. This assessment involved measuring the flux recovery following a 4 h photocatalytic cleaning process followed by a hydraulic cleaning stage, as shown in Figure 6. A higher flux recovery was observed in both membranes after the 4 h photocatalytic cleaning stage. Additionally, after the hydraulic cleaning, final flux recoveries of 94% and 92% were obtained for the UF-2P and MF-12P membranes, respectively. It also seems that the pore blocking fouling was more dominant in the UF membrane, as observed in Section 3.2.1. 

Additionally, the measurement of the photocatalytic efficiency of the UF-2P and MF-12P membranes in degrading an MB solution over the course of three cycles showed a minor variation in the performance of the membranes. Maximum changes of 6.5% for the UF-2P membrane and 5% for the MF-12P membrane were observed (Supplementary Materials, Figure S3). This indicates that the photocatalytic property of the membranes remains relatively consistent throughout the recycling process. MB, with the chemical formula C_16_H_18_ClN_3_S, is a cationic dye that dissolves in water and finds extensive application in the textile industry [54]. Various degradation pathways for MB have been determined. According to Houas et al., the degradation of MB in the presence of the TiO_2_ photocatalyst could occur via a decyclization and mineralization of molecules through continuous interactions with the hydroxyl radicals. As a result, the dye molecules are oxidized efficiently with an almost total mineralization of carbon, nitrogen, and sulfur heteroatoms, resulting in the formation of CO_2_, NH_4_^+^, NO_3_^−^, and SO_4_^2−^ [55]. 

#### 3.2.3. Contact Angle Measurement

The as-sprayed UF and MF membranes exhibit super hydrophilic surfaces with a contact angle of 0°. When a water droplet touches the surface of the as-sprayed membranes, it is instantly absorbed into the surface. The surfaces of UF-2P and MF-12P remained unchanged, with super hydrophilic properties after the HA self-cleaning process. However, the contact angle was slightly increased to 7° and 5.5° at the second and third cycles of photocatalytic degradation of MB for UF-2P. Likewise, increases to 3.3° and 7.5° were observed at the second and third cycles of photocatalytic degradation of MB for MF-12P. Despite this slight contact angle increase, the surface maintains its hydrophilic characteristics. In theory, hydrophilic membranes are considered to be less susceptible to fouling. The higher anti-fouling property could arise from the presence of hydrophilic OH species on the surface of TiO_2_ and the consequent water-shielding phenomenon [56,57]. However, as mentioned earlier, other parameters may also impact the fouling property of the membranes.

### 3.3. Infiltration of the UF Membranes with Agglomerates of TiO_2_ Nanoparticles (Filled Membranes)

As described in Section 3.2.1, the SPS UF exhibited a rather poor separation efficiency that could have been linked to the wide pore size distribution in those membranes. Thus, it was decided to investigate the possibility of improving the separation performance of the UF membranes by producing a narrower pore size distribution. Chung et al. [47] suggested a method to reduce the pore size distribution of the ceramic membranes by packing nanosized particles into the pre-existing pores of the membrane, following a calcination process to enhance the mechanical strength of the membranes. In this work, a similar approach was utilized to fill the larger pores in the UF-2P and UF-4P membranes. The heat treatment process was not carried out in our study to avoid potential oxidation and densification of the metallic substrate [58]. 

The as-sprayed UF-2P and UF-4P membranes were filtered with around 30 mL of 1 wt.% TiO_2_ (PiKem, Tamworth, UK) aqueous suspension to eliminate the large pores. The TiO_2_ powder used to fill the pores was identical to the powder used to fabricate the UF membranes in the SPS process with a particle size of 27 ± 10 nm and an agglomerate size of d_50_ = 3.1 µm, described in Section 2.1 [21]. The primary purpose was to decrease the pore size distribution of the membrane by embedding the agglomerates of nanosized TiO_2_, identical to those present in the microstructure of the suspension plasma-sprayed UF membranes, in the larger pores on the surface. The UF membranes filled with TiO_2_ nanoparticles have been referred to as filled membranes and are identified as UF-2P-F and UF-4P-F. 

#### 3.3.1. Surface Roughness

**Figure 7:**
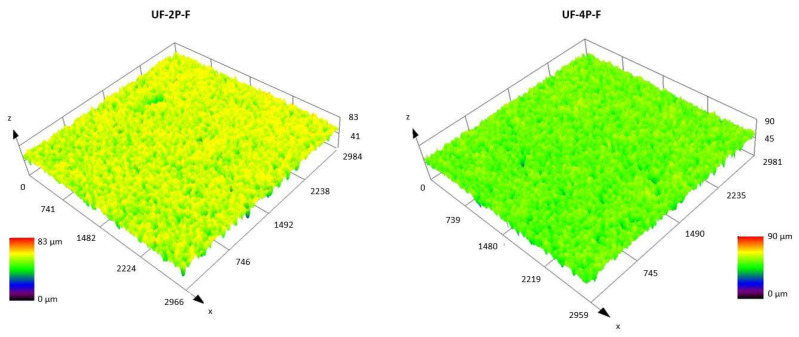
Confocal microscope image of UF-2P-F and UF-4P-F membranes.

Figure 7 shows the confocal microscope image of the surface UF-4P membrane after filling with TiO_2_ nanoparticles. Combined with the measurements seen in Table 5, it was observed that in both cases the R_a_ and R_z_ values were decreased due to filling up the pores on the surface of the membranes with TiO_2_ agglomerates. The decrease in the roughness is more significant in the UF-2P-F sample, which had a higher initial roughness. 

#### 3.3.2. Separation Performance

The UF-2P-F and UF-4P-F membranes were cleaned before rejection measurement. Following the filling of the membranes with TiO_2_ nanoparticles, the surface of the membranes was rinsed with DI water to remove the loose particles on the surface. After that, the membranes were compacted with DI water until obtaining the turbidity of 0 NTU for the filtrate. The compaction with DI water was performed to remove the residual loose agglomerates of nanoparticles.

**Figure 8:**
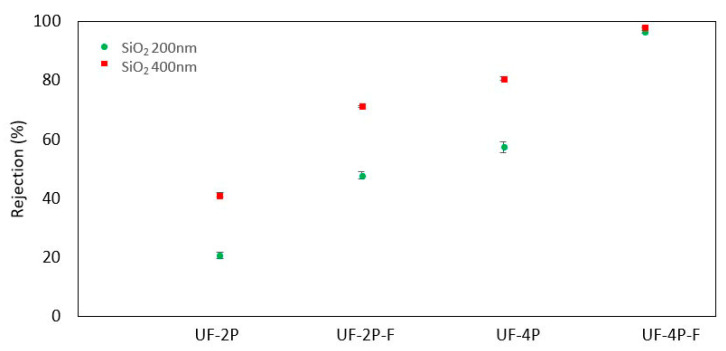
Comparing the rejection rates of the UF membranes before and after filling the large pores with agglomerates of TiO_2_ nanoparticles for SiO_2_ 200 nm and SiO_2_ 400 nm particles.

Figure 8 shows the particle rejection efficiency of the filled UF membranes. After filling the UF membranes with the agglomerates of TiO_2_ nanoparticles, the rejection rates of UF-2P-F and Uf-4P-F were increased compared to UF-2P and Uf-4P. Compared to UF-2P, for both 200 nm and 400 nm SiO_2_ particles, the rejection rates of the UF-2P-F membranes were improved by a factor of approximately two. On the other hand, the UF-4P-F membrane reached rejection rates of over 97% for 200 nm and 400 nm SiO_2_ particles, showing the importance of the membranes’ thickness on the separation efficiency. 

The membranes’ performance remained stable during the filtration process. However, the agglomerates of TiO_2_ nanoparticles were removed during the backwashing process, requiring a refilling process. 

#### 3.3.3. Molecular Weight Cut-Off (MWCO)

To further investigate the impact of incorporating TiO_2_ nanoparticles into the packing of ultrafiltration membranes to narrow the pore size distribution, the Molecular Weight Cut-Off (MWCO) of the UF-4P and UF-4P-F membranes was assessed. The evaluation of MWCO is a method to determine the rejection efficiency of UF membranes. The MWCO of the membranes corresponds to the molecular weight at which 90% rejection by the membrane was achieved [59]. 

**Figure 9:**
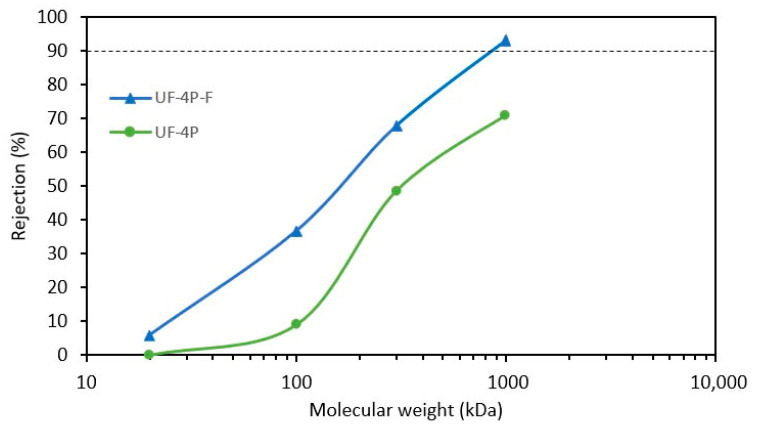
Rejection rate as a function of the PEO molecular weight of the UF-4P and UF-4P-F membranes. The filling process made it possible to reduce the MWCO of the filled membrane to about 900 kDa.

Figure 9 presents the MWCO measurements for UF-4P and UF-4P-F, indicating a decrease in the MWCO of the UF-4P-F. In this experiment, due to the range of PEO materials used, it was not possible to determine the 90% rejection point (MWCO) for the UF-4P sample (as-sprayed membrane). Since the as-sprayed UF membranes, especially at lower thickness, did not demonstrate favorable separation efficiency, higher-molecular-weight PEO materials were not employed to determine the MWCO of those membranes. Despite this limitation, the results clearly show that the MWCO of UF-4P is lower than that of UF-4P-F, which aligns with the particle rejection measurements of the as-sprayed and filled membranes. The MWCO of UF-4P-F was determined to be approximately 900 kDa, equivalent to a PEO molecule with a Stokes radius of roughly 33 nm. This suggests that the filled UF membrane could potentially remove molecules with a diameter of around 70 nm. However, it is worth noting that the MWCO is not an absolute determinant of the potential size of the particles that can be rejected since not all the foulant molecules and particles are spherical [59]. Furthermore, the shape of the pores could also influence the rejection efficiency [37,42]. In one case, the penetration of organic molecules over 30 times larger than the membrane pore size was reported by Arkhangelsky et al. [60].

The application of Equation (6) estimated the d_50_ of the UF-4P membrane to be approximately 37.5 nm, aligning with the average pore diameter of roughly 36 nm obtained using mercury intrusion porosimetry [21]. Furthermore, the estimated d_50_ of the UF-4P-F membrane was calculated to be about 29 nm. Filling the larger pores could lead to a sharper MWCO curve and improved rejection that could be due to a narrower pore size distribution [61]. Additionally, a stronger correlation was observed between the membranes’ pore size and the particle size of the TiO_2_ feedstock powder used in the SPS process to produce the membranes [21]. 

These results indicate that the filling process can improve the performance of the SPS UF membranes. However, optimizing the SPS process parameters is necessary to produce UF membranes with a narrow pore size distribution. An alternative approach could be to increase the thickness of the UF membranes.

## 4. Conclusions

This study evaluated the efficiency of TiO_2_ UF and MF membranes produced via the SPS process regarding their rejection rate, self-cleaning property, and recyclability. It was shown that

- The rejection efficiency of the MF and UF membranes can be tuned by adjusting the thickness of the membrane. Increasing the thickness of the membranes led to higher rejection rates, indicating the importance of membrane thickness in determining filtration performance.
- The SPS UF membrane exhibited enhanced rejection rates by effectively filling its larger pores with agglomerates of TiO_2_ nanoparticles. This modification improved the effective rejection of smaller particles, which could be attributed to the decrease in the average pore size and the total pore size distribution. This finding highlights the potential of incorporating nanoparticles to enhance the performance of ceramic membranes. Additionally, it suggests that the rejection efficiency of the membranes is influenced by both the average pore size and a uniform and narrow pore size distribution.
- Both SPS MF and UF membranes demonstrated recyclable self-cleaning properties under visible light, which is desirable for maintaining long-term filtration efficiency.
- Enhancing the performance of the UF membrane requires better optimization of its structure, focusing on achieving a more uniform membrane structure and narrow pore size distribution to enable more efficient filtration. The optimization can be carried out by adjusting the SPS process parameters.

Overall, the findings of this study provide valuable insights into the capabilities and limitations of the SPS process as a novel approach for manufacturing ceramic membranes. These understandings can be used to improve the structure and performance quality of SPS membrane technologies for various filtration applications. Furthermore, exploring the possible pathways to generate narrow pore size distribution in the SPS membranes by optimizing the SPS process parameters can be considered as a future task. Also, a comprehensive evaluation of the quality of the filtered water and optimization of the cleaning process can be conducted to provide a more thorough assessment of the performance efficiency of the membranes.

## Figures and Tables

**Table 1:** Identification of the membranes.

Sample Name	No. of Spray Passes
UF-2P	2
UF-4P	4
UF-2×4P	stacking two UF-4P
MF-12P	12
MF-2×12P	stacking two MF-12P

**Table 2:** Thickness measurements of the UF and MF membranes.

Sample Name	Thickness (µm)
UF-2P	17.2 ± 0.8
UF-4P	37 ± 1
MF-12P	20.5 ± 0.9

**Table 3:** The surface roughness of UF-2P, UF-4P, and MF-12P membranes.

Sample	Roughness (R_a_) (µm)	Roughness (R_z_) (µm)
UF-2P	5.3 ± 0.3	41 ± 2.6
UF-4P	3.4 ± 0.1	25.3 ± 2.2
MF-12P	6.4 ± 0.1	43.6 ± 1.2

**Table 4:** Flux recovery ratio (FRR %) of the UF and MF membranes.

Sample	FRR (%)	
	SiO_2_ (200 nm)	SiO_2_ (400 nm)
UF-2P	96.8	98.1
UF-4P	96.5	95.1
UF-2×4P	76.9	79.7
MF-12P	94.5	98.1
MF-2×12P	80.9	82.7

**Table 5:** Surface roughness of UF-2P-F and UF-4P-F. The membranes were filled with the agglomerates of nanosized TiO_2_.

Sample	Roughness (R_a_) (µm)	Roughness (R_z_) (µm)
UF-2P-F	3.5 ± 0.05	20.8 ± 0.4
UF-4P-F	2.9 ± 0.1	18.9 ± 0.7

## Data Availability

All data is in the manuscript.

## Supplementary Materials

The following supporting information can be downloaded at https://www.mdpi.com/article/10.3390/membranes13090750/s1. Figure S1: Particle size distribution of the two SiO_2_ particles used for characterizing the efficiency of the particle rejection in the membranes showing (a) the particle size distribution of SiO_2_ powder with the average particle size of 200 nm, and (b) the particle size distribution of the SiO_2_ powder with the average particle size of 400 nm; Figure S2: Normalized flux during the SiO_2_ separation process for UF and MF membranes; Figure S3: Recyclability of UF-2P and MF-12P in terms of the photocatalytic degradation of MB, showing relatively consistent performance. References [62,63,64] are cited in the supplementary materials.
