# Supplementary material for: A Comparative Study of the Self-Cleaning and Filtration Performance of Suspension Plasma-Sprayed TiO2 Ultrafiltration and Microfiltration Membranes
Source: Membranes (Basel). 2023 Aug 23;13(9):750. doi: 10.3390/membranes13090750 (PMC10534907; doi:10.3390/membranes13090750)
Supplement: Supplementary file 1 [file membranes-13-00750-s001.zip › membranes-2549230-supplementary.pdf]

Supplementary Materials

A Comparative Study of the Self-cleaning and Filtration Performance of Suspension Plasma Sprayed TiO_2 Ultrafiltration and Microfiltration Membranes

Elnaz Alebrahim[1*], Christian Moreau[1]

[1] Department of Mechanical, Industrial, and Aerospace Engineering, Concordia University, 1455 de Maisonneuve Blvd. W, Montreal, H3G 1M8, Quebec, Canada

* elnazsadat.aleebrahim@mail.concordia.ca

This document contains supplementary information related to the article " A Comparative Study of the Self-cleaning and Filtration Performance of Suspension Plasma Sprayed TiO2 Ultrafiltration and Microfiltration Membranes". The below-mentioned sections have been labeled in correspondence with the sections in the main manuscript, to provide easy navigation and quick access to relevant content**.**

2.1. Membrane Preparation (Brief Description of the SPS Membranes According to Previous Works)

In our previous works, the suspension plasma spray (SPS) process was used to produce membranes with pore sizes in the range of MF and UF. It was observed that in both UF and MF structures, there existed a significant correlation between the particle size of the feedstock material and the resulting average pore size in the membranes. Moreover, it was described that in the SPS membranes, the porosity mainly depended on the space amongst the unmelted inflight particles trapped and surrounded by the microstructure formed from fully melted particles. Hence, the correlation between the feedstock particle size and membrane pore size was attributed to the presence of unmelted feedstock particles within the structure [1,2].

Generally, a high surface roughness is undesirable for membrane application since it could increase membrane fouling [3]. In SPS coatings, columnar features forming bumps on the top surface may develop by the deflection of the smaller inflight particles close to the surface of the substrate during the SPS process. As a result, the coating is built by attaching the deviated particles to the sides of the asperities on the surface of the substrate. As the thickness of the coating increases, bumps may appear on the surface of the coatings, increasing the surface roughness [4]. In the SPS process, the formation of these bumps is influenced by the roughness of the substrate and the size of the feedstock particles. Higher surface roughness and smaller feedstock size could increase the likelihood of the occurrence of this phenomenon [5]. In our previous works, the columnar features were observed only on the surface of the MF membranes, produced with submicron-sized feedstock inflight [2]. On the other hand, in the UF membranes, bumps on the surface were less intense [1]. Although in the UF membrane, the individual particles of the feedstock were around 30 nm, they formed a few micron-sized agglomerates that were less subjected to the drag forces of the plasma [6]. Thus, in the UF membrane, a preferential filling of the pores on the substrate occurred, and less roughness was observed compared to the MF membrane [1,2]. Furthermore, by increasing the thickness of the UF membranes, the surface becomes less susceptible to the form of asperities on the surface of the substrate resulting in lower roughness. On the other hand, in the MF membrane, where the feedstock powder is much smaller with a d_{50} of around 280 nm, a rougher coating surface was obtained.

2.3. Membrane Performance

Figure S1 shows the particle size distribution of the SiO_2 particles used for the rejection efficiency measurements.

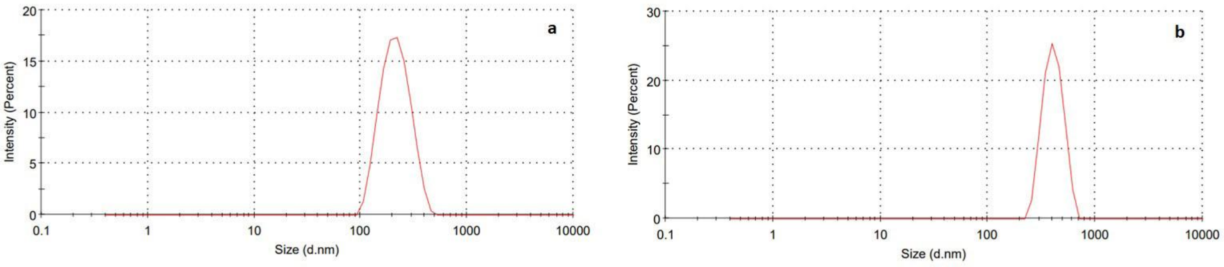

Figure S1 Particle size distribution of the two SiO_2 particles used for characterizing the efficiency of the particle rejection in the membranes showing a) the particle size distribution of SiO_2 powder with the average particle size of 200 nm, and b) the particle size distribution of the SiO_2 powder with the average particle size of 400 nm.

3.2.1. Separation Performance

Figure S2 presents the normalized flux of the UF and MF membranes during the SiO_2 separation process.

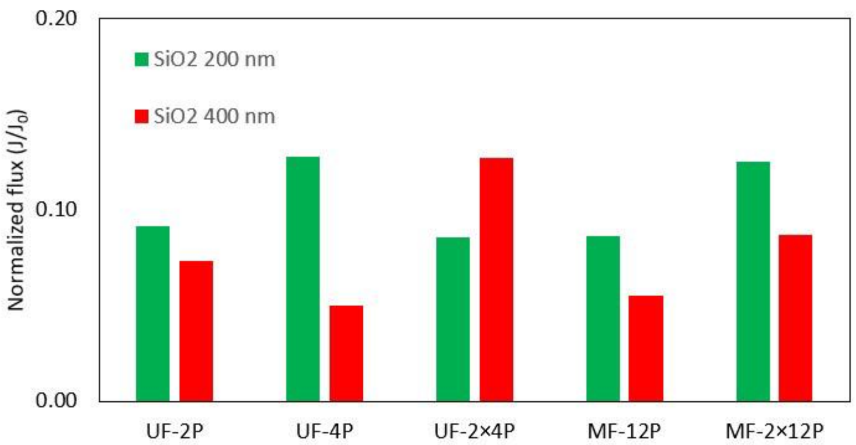

Figure S2 Normalized flux during the SiO_2 separation process for UF and MF membranes

3.2.2. Self-cleaning and recyclability

Figure S3 shows the photocatalytic efficiency of the UF-2P and MF-12P membranes in degrading an MB solution over the course of 3 cycles.

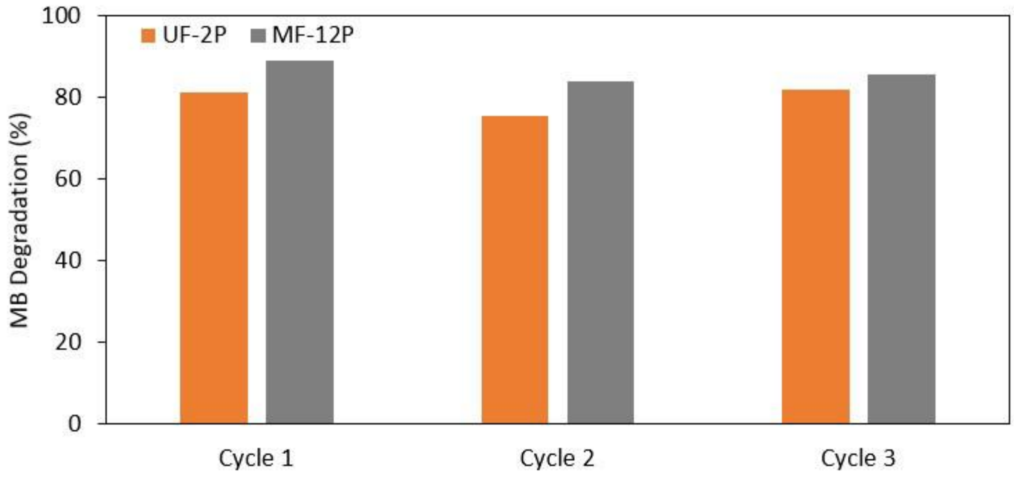

Figure S3 Recyclability of UF-2P and MF-12P in terms of the photocatalytic degradation of MB, showing relatively consistent performance.

Reference

[1] E. Alebrahim, M.S. Rahaman, C. Moreau, TiO2 Photocatalytic Ultrafiltration Membrane Developed with Suspension Plasma Spray Process, Coatings. 12 (2022) 1764. https://doi.org/10.3390/coatings12111764.

[2] E. Alebrahim, F. Tarasi, Md.S. Rahaman, A. Dolatabadi, C. Moreau, Fabrication of titanium dioxide filtration membrane using suspension plasma spray process, Surf Coat Technol. 378 (2019) 124927. https://doi.org/10.1016/j.surfcoat.2019.124927.

[3] K.C. Khulbe, C.Y. Feng, T. Matsuura, Synthetic Polymeric Membranes, Springer Berlin Heidelberg, Berlin, 2008.

[4] G. Mauer, C. Moreau, Process Diagnostics and Control in Thermal Spray, Journal of Thermal Spray Technology. 31 (2022) 818–828. https://doi.org/10.1007/s11666-022-01341-z.

[5] G. Mauer, R. Vaßen, Coatings with Columnar Microstructures for Thermal Barrier Applications, Adv Eng Mater. 22 (2020) 1–9. https://doi.org/10.1002/adem.201900988.

[6] J. Oberste Berghaus, S. Bouaricha, J.G. Legoux, C. Moreau, Injection conditions and in-flight particle states in suspension plasma spraying of alumina and zirconia nano-ceramics, in: The International Thermal Spray Conference (ITSC) Basel, Switzerland, 2005: pp. 1434–1440.
